# Transcriptional Regulation of Brassinosteroid Accumulation during Carrot Development and the Potential Role of Brassinosteroids in Petiole Elongation

**DOI:** 10.3389/fpls.2017.01356

**Published:** 2017-08-11

**Authors:** Feng Que, Guang-Long Wang, Zhi-Sheng Xu, Feng Wang, Ai-Sheng Xiong

**Affiliations:** State Key Laboratory of Crop Genetics and Germplasm Enhancement, College of Horticulture, Nanjing Agricultural University Nanjing, China

**Keywords:** Brassinosteroids, biosynthesis, signal transduction, gene regulation, 24-epibrassinolide, *Daucus carota* L.

## Abstract

It is widely known that brassinosteroids (BRs) are involved in various physiological processes during plant growth and development. Roles of BRs have been reported in many plants. However, relevant report is yet not found in carrot. Carrot is a nutrient-rich vegetable from the *Apiaceae* family. Here, we measured the bioactive contents of BRs at five successive stages and analyzed the expression profiles of genes involved in BR biosynthesis, signaling pathway and catabolism. We found that most biosynthesis regulated genes had higher expression level at the first development stage of carrot and the catabolism gene *BAS1*/*CYP734A1* had significantly high expression level at the first stage in carrot roots and petioles. In addition, we treated carrot plants with exogenous 24-epibrassinolide (24-EBL) and examined the morphological changes after treating. Compared with control plants, carrot plants treated with 24-EBL had higher plant height, more number of petioles and heavier aboveground weight. The expression levels of *DcBRI1, DcBZR1*, and *DcBSU1* in the petioles were significantly up-regulated by treating with exogenous 24-EBL. The expression profiles of *DcCYP734A1* were all significantly up-regulated in the three organs when treated with 0.5 mg/L 24-EBL. The elongation of carrot petioles can be promoted by treating with exogenous 24-EBL. These results indicate that BRs playing potential roles during the growth and development of carrot.

## Introduction

Brassinosteroids (BRs), a kind of sterols that were first discovered and isolated from *Brassica napus* pollen, have been reported to be involved in many aspects of plant growth and development (Grove et al., 1979; Clouse, 1997). In the growth and development of plant roots, BRs were reported to participate in maintenance of meristem size, lateral root initiation, nodulation in legume species and root hair formation (Bao et al., 2004; Terakado et al., 2005; Hacham et al., 2011; Cheng et al., 2014). Moreover, the roots of BR deficient mutant were found significantly shortened (Müssig et al., 2003). In *Arabidopsis*, BR was found working cooperatively with auxin in promoting petiole elongation to response the shade stimulus (Kozuka et al., 2010). BRs were also reported to be involved in stem elongation, cell division, vascular differentiation, and leaf bending and epinasty (Thompson et al., 1982; Clouse et al., 1996; Tong and Chu, 2012; Hao et al., 2013). In field production, BRs are believed to improve crop yield and plant tolerance against environmental stress (Divi and Krishna, 2009).

To date, more than 70 kinds of BRs have been identified (Miransari, 2014). Among them, brassinolide (BL) is recognized as the most active form (Fujioka and Sakurai, 1997). With the rapid development of biotechnology, the biosynthesis and signaling pathways of this hormone have been well studied. BL is produced *via* the early C-6 oxidation pathway and late C-6 oxidation pathway (Noguchi, 2000). Numerous studies have shown that regulation of genes involved in BR biosynthesis pathway can result in alteration in BR accumulation and plant growth (Yoshimitsu et al., 2011; Zhiponova et al., 2013). Some genes, such as *DWARF7* (*DWF7*), *DWARF1* (*DWF1*), *DE ETIOLATED2* (*DET2*), *DWARF4* (*DWF4*), *CONSTITUTIVE PHOTOMORPHOGENESIS AND DWARFISM* (*CPD*), *CYP85A2*, and *CYP90C1* have been verified to play roles in BR biosynthesis (Choe et al., 1998, 1999; Noguchi, 1999; Du and Poovaiah, 2005; Ohnishi et al., 2006, 2012; Nole-Wilson et al., 2010). Among them, *DWF4* is reported to be a key gene in BR biosynthesis, which may control the putative rate-limiting step in the BR biosynthetic pathway (Choe et al., 1998, 2001).

The homeostasis of BRs is regulated by biosynthesis genes and catabolism genes (Tanaka and Okamoto, 2005). In *Arabidopsis, BAS1/CYP734A1* is a major BR inactivating gene (Neff et al., 1996). In many higher plants, the *CYP734A* paralogs was also found (Thornton et al., 2011). In addition, genes involved in the signaling pathway of BRs have been well investigated in *Arabidopsis*. Brassinosteroid insensitive 1 (BRI1), BRI1-associated receptor kinase1 (BAK1), BR-signaling kinase1 (BSK1), BRI1 suppressor 1 (BSU1), bridging integrator 2 (BIN2), brassinazole resistant1 (BZR1), and BRI1-EMS-suppressor1 (BES1) were involved in the signaling pathway (Wang and He, 2004; Sun et al., 2010; Yan et al., 2012; Shi et al., 2013a,b; Zhang et al., 2014; Jiang et al., 2015). Among these genes, *BRI1* works as the receptor for BRs, *BZR1*and *BES1* are the transcription factors that regulate the growth of plants (Gallegobartolomé et al., 2012; Guo et al., 2013).

Numerous studies have been conducted to determine the function of BRs in plant growth. However, BR accumulation and its potential roles during carrot growth and development remain elusive. Carrot (*Daucus carota* L.) is a nutrient-rich root crop from the *Apiaceae* family (Luby et al., 2014; Xu et al., 2014; Wang et al., 2015). It is one of the most economically important members of *Apiaceae* plants, and the cultivated area of carrot is progressively increasing worldwide (Cavagnaro et al., 2011).

In this study, BR accumulation at five successive growth stages was examined in carrot. Expression profiles of genes involved in BR biosynthesis and signaling pathways were also analyzed. Besides, exogenous 24-epibrassinolide (24-EBL) was applied to carrot plants to study the putative effects of BR on carrot. The results of our work would shed novel insights into studies focusing on hormonal control of plant growth.

## Materials and Methods

### Plant Material and Exogenous 24-EBL Treatment

‘Kurodagosun’ was selected as the experimental material and cultivated in a climate-control chamber at Nanjing Agricultural University (32°04′N, 118°85′E). Ambient temperature was held at 25°C for 16 h during daytime and 18°C for 8 h in the dark. The mix vermiculite and organic soil (1:1, v/v) was used to cultivated plants. The carrot samples were collected at 20 (stage 1), 40 (stage 2), 60 (stage 3), 75 (stage 4), and 90 (stage 5) days after sowing (DAS). The five stages were classified based on dates and morphological characteristics. The roots, petioles, and leaf blades were separately collected at different stages, and stored at -80°C for molecular research.

To investigate the potential effect of BR on carrot, 40 DAS carrot plants were treated with 24-EBL. The 24-EBL (≥ 85 %, Sigma–Aldrich) was dissolved in ethanol and then diluted by distilled water to make mother liquor. Four concentrations of exogenous 24-EBL (0, 0.1, 0.5, and 1 mg/L) were used, respectively. Plants treated with water alone were used as control (CK). Treatment was carried out every 2 days, five times in total. Afterward, the plants were allowed to grow for another 20 days and harvested for morphology determination.

### Assay of Bioactive BR Levels

Samples were ground in a mortar with 10 mL of 80% methanol extraction solution containing 1 mM butylated hydroxytoluene. The mixture was incubated for 4 h at 4°C. The samples were then centrifuged for 10 min at 3500 *g*. The supernatants were filtered through a C_18_-Sep-Pak cartridge (Waters, Milford, MA, United States), and the efflux was collected and dried with N2. The mixture was dissolved in 2 mL of PBS containing 0.1 % (v/v) Tween 20 and 0.1% (w/v) gelatin (pH 7.5). The samples were analyzed *via* indirect enzyme-linked immunosorbent assay according to the methods described previously (Swaczynov et al., 2007; Pradko et al., 2014). Briefly: (1) The calibrating samples (epibrassinolide, CAS: 72962-43-7) or test samples (150 μL per well) were put in wells of the plate with the immobilized antibodies. Plates were placed at 37°C for 30 min. Then, removed the liquid from the wells and washed plates four times with washing buffer. (2) The Horseradish peroxidase (HRP)-conjugate (150 μL) was placed in the wells and placed at 37°C for 30 min. Then, removed the liquid from the wells and washed plates four times with washing buffer. (3) Added TMB solution (containing H_2_O_2_) to the wells and placed the plates at 37°C for 20 min. (4) Quenched the reaction by adding 2 mol/L H_2_SO_4_ (50 μL) into each well. (5) Measured optical absorbance at 450 nm. (6) Calculated the concentration according to the calibration curve. The epibrassinolide and other related brassinolide analogs were measured in this study.

### Total RNA Isolation

An RNA extraction kit (Tiangen, Beijing, China) was used to extract the total RNA of carrot roots, leaf blades, and petioles according to the manufacturer’s instructions. cDNA was synthesized using a PrimerScript RT reagent kit (TaKaRa, Dalian, China). The cDNA was diluted 15-fold for quantitative reverse transcription PCR (qRT-PCR) analysis.

### qRT-PCR Analysis

Genes involved in BR biosynthesis and signal transduction pathway were selected from CarrotDB^1^ (Xu, 2014). The primers of each gene were designed via Primer Premier 5.0 software, and were displayed in **Table 1**. qRT-PCR was performed in a real-time PCR detection system (Bio-Rad, Hercules, CA, United States). The cycling conditions were maintained as follows: 94°C for 30 s, 40 cycles at 94°C for 10 s, 58°C for 20 s, and 61 cycles at 65°C for 10 s to create a melting curve. The experiments were performed with three independent biological replicates. Under normal growing condition, the *DcACTIN* gene was selected as the internal control to analyze gene expression and data of *DcDWF4* in carrot petioles at 90 DAS were chosen as a calibrator for gene expression analysis (Tian et al., 2015). By contrast, in the analysis of gene expression after application of 24-EBL, the *DcTUB* gene was selected as the internal control. The expression of *DcTUB* gene was more stable under abnormal growth conditions.

**Table 1:** Oligonucleotide sequences of genes involved in the biosynthesis, catabolism, and signaling pathway.

Gene name	Oligonucleotide sequences (5′→3′)
*DcDWF4*	Forward primer: AAACGCTAAGGCTGGGCAATGT
	Reverse primer: GCACGGCTGCAATCACTGGAA
*DcCPD*	Forward primer: TCCCACCTACCGTAAAGCCATT
	Reverse primer: ATCATTCTTCCTCTCCCCTCCT
*DcDET2*	Forward primer: TCAACGCCTCTCTCCTCACTCT
	Reverse primer: CCAGCCCTTACGGTGGTGTTT
*DcCYP90C1*	Forward primer: GGTATGGCGAAAACAGGAGAGA
	Reverse primer: AACACGAGATAGAGTGGCAGGG
*DcCYP85A2*	Forward primer: AGCCACTTACATTTAATCCCTG
	Reverse primer: TCTCCTCCAACTTCTTCCCATC
*DcDWF5*	Forward primer: AGATGGTGGTGAAGGAGGAGAA
	Reverse primer: CACGCAGTCATAGTGGGTTTTG
*DcDWF1*	Forward primer: TCCGACCTTTTCTACGCTATTC
	Reverse primer: TGTAACCCTGTGCCAGTTCTTT
*DcBAK1*	Forward primer: TAGCACCCGAGTACCTATCCAC
	Reverse primer: AGCAACATCACATCGTCATCAT
*DcBRI1*	Forward primer: GAAAAGGAGGAAGAAGAAGGAA
	Reverse primer: CAAGAAGATCTGCAAAAGTGAG
*DcBSK1*	Forward primer: TGGCTGCTCTAGCTTAGTCTCC
	Reverse primer: TTCCTTAGTTGCTCCAATGTGT
*DcBZR1*	Forward primer: AGTTTCCAGCCATCGCCGTCCC
	Reverse primer: CTGCTCCCCAGCCCAATCCTCC
*DcBSU1*	Forward primer: TCAGCTTTTTAACTATCTTCCA
	Reverse primer: ACCTTCTACACTATCATTTTCT
*DcBIN2*	Forward primer: TTGTTCCCACTGTTTCTTTGATG
	Reverse primer: TGTAGATAGCCCACTTTGTCTCC
*DcACTIN*	Forward primer: AGAAGCACCACTGAATCCTAAGGC
	Reverse primer: GCATACACCATCACCAGAGTCCAA
*DcTUB*	Forward primer: CGGTATTGTGTTGGACTCTGGTGAT
	Reverse primer: CAGCAAGGTCAAGACGGAGTATGG
*DcCYP734A1*	Forward primer: TGCCATTCAGCCTTGGAGTA
	Reverse primer: TGCGGATAAAGAAGCATCAACACC

### Statistical Analysis

Difference in the BRs bioactive content and the expression levels of genes in different organs during carrot development were detected by Duncan’s multiple-range test at a 0.05 probability level. Student’s *t*-test was used to identify the differences under different concentration treatments at the 0.05 significance.

## Results

### Plant Growth Analysis

**FIGURE 1:**
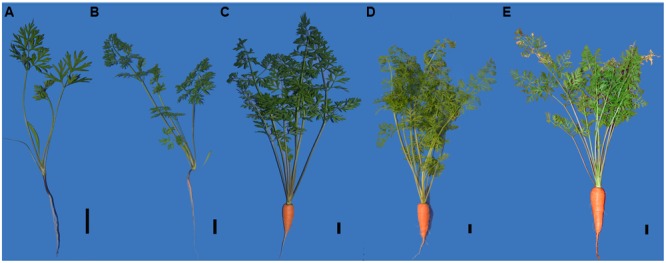
Growth status of carrots from five different developmental stages in this research. **(A)** Stage1, 20 days after sowing, **(B)** stage2, 40 days after sowing, **(C)** stage3, 60 days after sowing, **(D)** stage4, 75 days after sowing, **(E)** stage5, 90 days after sowing. Black lines in the lower right corner of each plant represent 2 cm in that pixel.

The plant materials were sampled at five stages (20–, 40–, 60–, 75–, and 90 DAS) (**Figure 1**). The root was white at the first stage, and turned orange at the second stage. The weight of roots, petioles, and leaf blades remarkably increased between stages 2 to 3. The root continued to enlarge, and this trend was maintained at the remaining stages.

### Changes in Bioactive BR Contents

**FIGURE 2:**
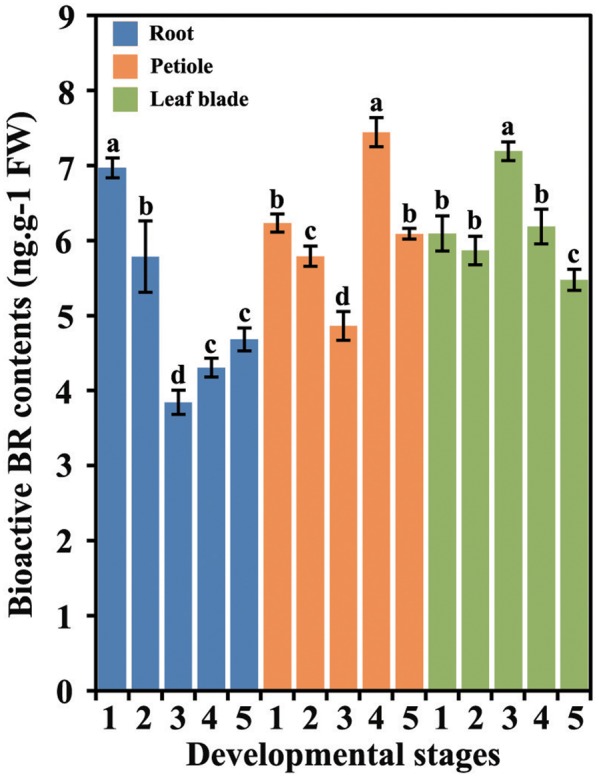
Bioactive BR levels in different tissues during carrot growth and development. Error bars represent standard deviation (SD). Columns with the same letter are not significantly different (*P* < 0.05).

The levels of bioactive BRs in different tissues at five different stages were measured (**Figure 2**). BR accumulation differed in different carrot tissues and at different developmental stages.

In the roots, the levels of bioactive BRs were highest at first stage followed by a decrease until stage 3. The BRs levels underwent a slight increase at stage 4 and were constant until the last stage. In the petioles, the highest level of BRs was detected at 75 DAS (stage 4), whereas the lowest level was found at 60 DAS (stage 3). In the leaf blades, the level of BRs was peaked at the third stage.

### Effects of 24-EBL on the Growth and Development of Carrot

**FIGURE 3:**
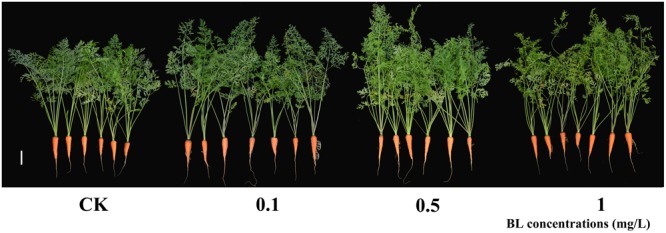
Growth status of carrots treated with exogenous BL. 40 days after sowing plants were treated with different concentrations of BL (0, 0.1, 0.5, and 1 mg/L). White lines in the lower right corner of each plant represent 5 cm.

**FIGURE 4:**
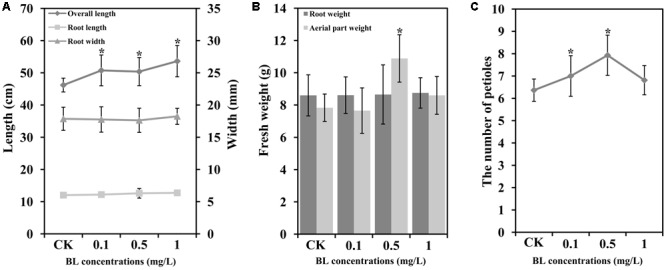
Exogenous BL induces petiole growth in carrot. 40 days after sowing plants were treated with different concentrations of BL (0, 0.1, 0.5 and 1 mg/L). **(A)** Root length, overall length, and root width; **(B)** root weight and aerial part (petioles and leaf blades) weight; **(C)** the number of petioles. Student’s *t*-test was used to identify the differences under different treatments (*P* < 0.05; ^∗^, control versus treatment). Error bars represent standard deviation (SD).

To determine the potential roles of BR during the development of carrot, 40 DAS (stage 2) carrot plants were treated with four different concentrations of exogenous 24-EBL (0, 0.1, 0.5, and 1 mg/L) (**Figure 3**). Six physiological indexes including total plant height, root length, root diameter, root weight, aboveground weight, and number of petioles were selected for comparison among the treatments. Under different concentrations of 24-EBL treatments, the root length, root diameter, and root weight did not show obvious difference compared with control. However, 24-EBL obviously increased the total plant height of carrot (**Figures 4A,B**). When carrot plants were treated with 0.5 mg/L 24-EBL, the aboveground weight of carrot was significantly increased compared to the control (**Figure 4B**). Similarly, carrot plants exposed to 0.5 mg/L 24-EBL displayed increased number of petioles when compared with control plants (**Figure 4C**).

### Expression Profiles of Genes Involved in BR Biosynthesis and Catabolism

**FIGURE 5:**
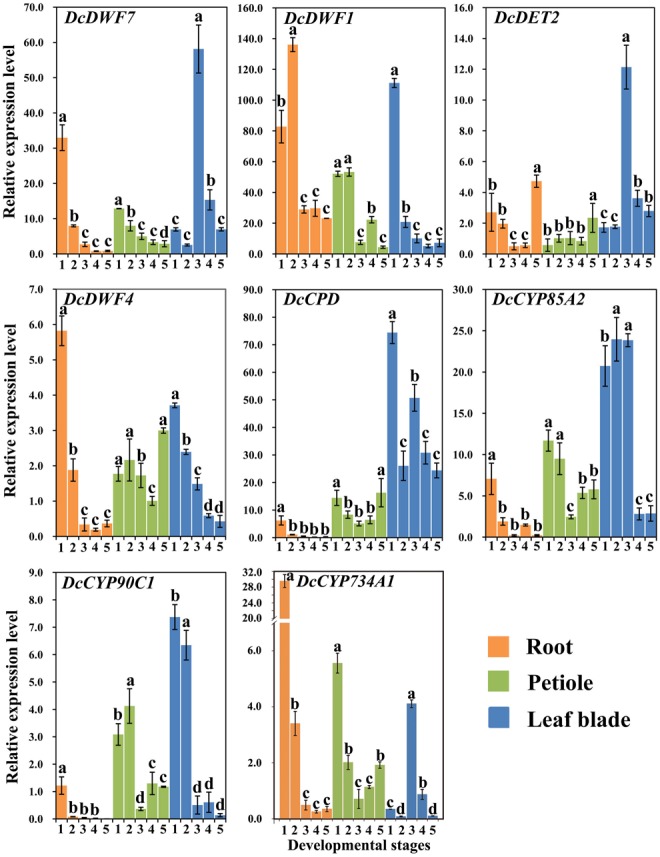
Expression profiles of genes involved in the BR biosynthesis and catabolism pathway at different stages and in different organs. Error bars represent standard deviation (SD). Columns with the same letter are not significantly different (*P* < 0.05).

*DcDWF7, DcDWF1, DcDET2, DcDWF4, DcCPD, DcCYP85A2, DcCYP90C1*, and *DcCYP734A1* were recognized as genes involved in BR biosynthesis and catabolism and their expression levels in carrot roots, petioles and leaf blades at five developmental stages were measured by qRT-PCR (**Figure 5**) (Noguchi, 2000; Choe, 2010).

#### In the Roots

*DcDWF4*, a key gene in BR biosynthesis, showed the highest expression level at stage 1 and the lowest level at stage 4. *DcCPD* and *DcCYP90C1* peaked at stage 1 followed by a decrease. Afterward, the highest level of *DcDET2* appeared at stage 5. *DcDWF1* showed higher expression as compared with other genes. *DcCYP734A1* is a catabolism gene of BRs and showed significantly higher expression level at stage 1 compared with other stages.

#### In the Petioles

The minimum expression level of the five genes (i.e., *DcDWF4, DcCPD, DcDET2, DcCYP90C1*, and *DcCYP85A2*) was observed at stage 3 or 4. The five genes exhibited similar expression trends. *DcDWF7* and *DcDWF1* were lowly expressed at the last stage. Transcription level of *DcDWF1* was relatively higher than that of others. The expression level of catabolism gene *DcCYP734A1* peaked at stage 1.

#### In the Leaf Blades

*DcDWF7, DcDET2*, and *DcCYP85A2* were highly expressed at third stage. *DcDWF1, DcDWF4*, and *DcCYP90C1* showed a similar expression pattern which peaked at first stage and gradually decreased afterwards. In the leaf blades, *DcCYP734A1* gene showed the highest expression level at stage 3.

### Expression Profiles of Genes Involved in the BR Signaling Pathway

**FIGURE 6:**
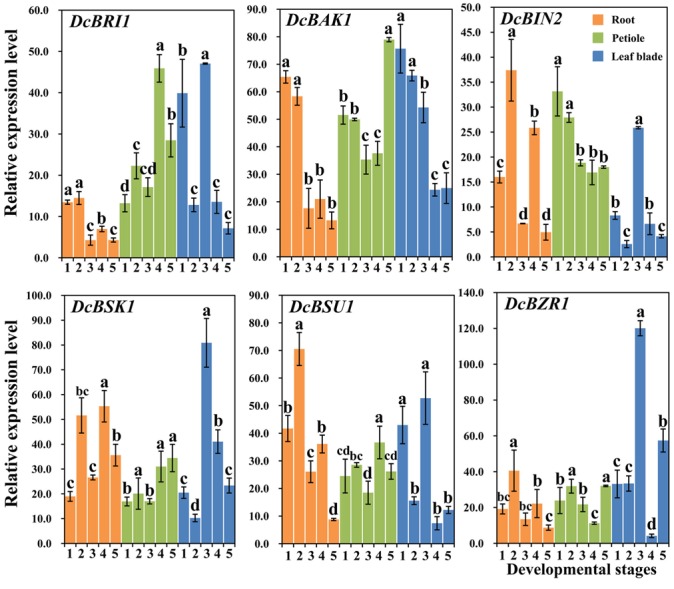
Expression profiles of genes involved in the BR signaling pathway at different stages and in different organs. Error bars represent standard deviation (SD). Columns with the same letter are not significantly different (*P* < 0.05).

In this study, transcript levels of *DcBAK1, DcBRI1, DcBSK1, DcBZR1, DcBSU1*, and *DcBIN2*, which have been reported to be involved in the BR signaling pathway, were analyzed using qRT-PCR (Belkhadir et al., 2006; Wang et al., 2006; Gendron and Wang, 2007). The expression profiles are shown in **Figure 6**.

#### In the Roots

*DcBRI1* and *DcBAK1* showed higher expression levels at the first two stages and relatively lower expression levels at later stages. The expression levels of *DcBSK1, DcBIN2, DcBSU1*, and *DcBZR1* increased at second stage and declined at the third stage, and then increased at stage 4 and decreased at stage 5.

#### In the Petioles

*DcBRI1* and *DcBSU1* were highly expressed at the fourth stage, whereas the lowest expression level of *DcBZR1* was detected at this stage. *DcBAK1, DcBSK1, DcBZR1* showed highest expression level at the last stage. The highest transcription of *DcBIN2* was observed at the first stage, followed by a continuous decrease at the remaining stages.

#### In the Leaf Blades

*DcBRI1, DcBSK1, DcBSU1, DcBZR1*, and *DcBIN2* showed higher expression levels at stage 3. *DcBAK1* showed relatively stable expression level at the first two stages, followed by a obvious decrease at the later stages.

### Effects of 24-EBL Application on the Expression Level of BR Biosynthetic and Catabolism Genes

**FIGURE 7:**
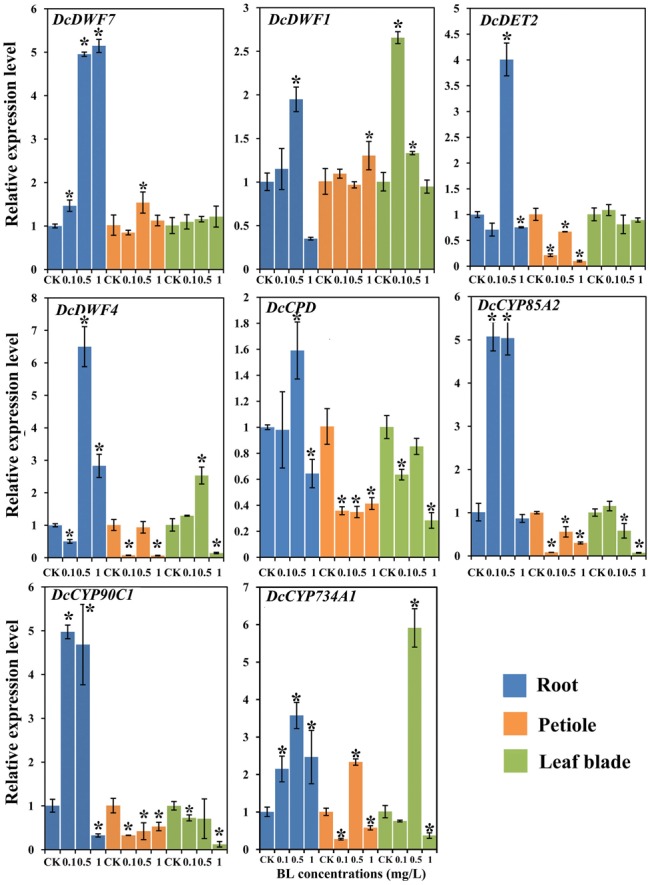
Expression profiles of genes involved in the BR biosynthesis and catabolism pathway under different BL concentrations (0, 0.1, 0.5, and 1 mg/L) and in different organs. Error bars represent standard deviation (SD). Student’s *t*-test was used to identify the differences under different concentration treatments (*P* < 0.05; ^∗^, control versus treatment).

To study the effect of 24-EBL on BR biosynthesis, we investigated the expression level of related genes. The expression levels of these genes under different BL concentrations were measured by quantitative reverse transcription PCR (qRT-PCR). These genes were strongly regulated by exogenous BL treatment (**Figure 7**).

#### In the Roots

All the genes examined were significantly up-regulated at the 0.5 mg/L. In the presence of 1 mg/L 24-EBL, *DcDWF1, DcCPD*, and *DcCP90C1* showed lower expression levels as compared with control plants. When compared with control group, *DcDWF4* expression level was significantly reduced in the roots treated with 0.1 mg/L BL, whereas transcription of *DcCYP85A2* and *DcCYP90C1* was increased.

#### In the Petioles

The expression levels of *DcDET2, DcDWF4, DcCPD, DcCYP85A2*, and *DcCYP90C1* were significantly down-regulated after application of exogenous BL. In the expression profile of *DcDWF7*, expression level did not show significant change with 0.1 and 1 mg/L 24-EBL, but the expression level was significantly up-regulated with 0.5 mg/L. The catabolism gene *DcCYP734A1* was significantly up-regulated under the treatment of 0.5 mg/L 24-EBL.

#### In the Leaf Blades

The expression levels of *DcCPD, DcCYP90C1*, and *DcCYP85A2* was down-regulated after application of 24-EBL. Meanwhile, the expression levels of *DcDWF7* and *DcDET2* did not show significant change. However, *DcDWF1* showed up-regulation trend with 0.1 and 0.5 mg/L and *DcDWF4* was up-regulated by 0.5 mg/L 24-EBL. The expression level of the catabolism gene *DcCYP734A1* was significantly up-regulated under the treatment of 0.5 mg/L 24-EBL.

### Effects of 24-EBL Treatment on the Expression Profiles of BR Response Genes

**FIGURE 8:**
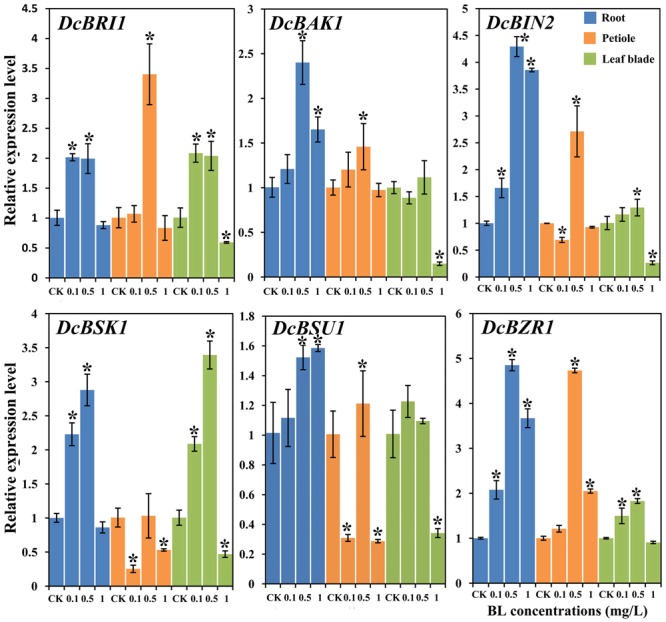
Expression profiles of genes involved in the BR signaling pathway under different BL concentrations (0, 0.1, 0.5, and 1 mg/L) and in different organs. Error bars represent standard deviation (SD). Student’s *t*-test was used to identify the differences under different concentration treatments (*P* < 0.05; ^∗^, control versus treatment).

To further investigate the effect of 24-EBL application on the expression levels of BR response genes, six genes were selected and their expression profiles were evaluated by qRT-PCR. Most genes were strongly regulated after the treatment (**Figure 8**).

#### In the Roots

The expression levels of all genes was increased in the presence of 0.5 mg/L BL. *DcBRI1*, and *DcBSK1* expression levels was up-regulated in the plants treated with 0.1 and 0.5 mg/L BL. However, their expression levels in the presence of 1.0 mg/L showed no significant difference as compared with control group. *DcBAK1, DcBIN2, DcBSU1*, and *DcBZR1* were up-regulated at all the three concentrations 24-EBL levels.

#### In the Petioles

Most genes showed significant up-regulation at the concentration of 0.5 mg/L, whereas *DcBSK1* expression level showed no significant change. The expression levels of *DcBSK1* and *DcBSU1* was down-regulated when treated with 0.1 and 1 mg/L. Transcription of *DcBZR1* was significantly increased in the presence of 1 mg/L BL.

#### In the Leaf Blades

Most genes (except *DcBAK1* and *DcBSU1*) were up-regulated in the presence of 0.5 mg/L BL. When treated with 1 mg/L BL, the expression level of most genes significantly decreased, whereas *DcBZR1* showed no obvious change. *DcBAK1* and *DcBIN2* expression levels in plants treated with 0.1 and 0.5 mg/L BL did not differ significantly from control.

## Discussion

Phytohormones are very important factors affecting almost all the processes of plant growth (Singh and Savaldigoldstein, 2015). Brassinosteroids are a class of phytohormones with various functions in plants. They have been reported to play important roles in many developmental processes including primary root extension and lateral root formation, seed germination, cell and stem elongation, abiotic stress responses (Roddick and Guan, 1991; Clouse et al., 1996; Li and Chory, 1997; Bao et al., 2004; Sharma et al., 2015). Functions of BRs have been documented in many plants, such as, tomato, cucumber, rice, and maize (Montoya et al., 2005; Fu et al., 2008; Kir et al., 2015). However, relevant study regarding BRs in carrot is rare.

The precise role of a specific hormone on plant growth and development is not only a result of its available levels, but also relates to the plant species, and the target organ (Singh and Savaldigoldstein, 2015). In addition, the roles of a specific hormone also depend on the presence of other phytohormone and plant growth regulators (Nemhauser et al., 2006). BRs, acting as a growth-promoting hormone were reported to be most actively biosynthesized in actively growing tissues and function at the sites of synthesis. In *Arabidopsis*, the highest and the second highest level of endogenous BRs were observed in apical shoots and developing siliques which were actively developing organs, respectively (Shimada et al., 2003; Montoya et al., 2005).

In carrots, the content of bioactive BR levels at different stages and in different organs keeps changing (**Figure 2**). These results suggested that the roles of BRs were different at different developing stages. At stage1, the main function of BRs may be promoting the formation of roots. At stages 3 and 4, BRs may mainly promote the development of leaf blades and petioles, respectively. Here, the content of bioactive BRs was detected by ELISA. ELISA is an indirect approach for measuring BRs. According to previous reports, high performance liquid chromatography (HPLC), high performance liquid chromatography-mass spectrometry (HPLC-MS), and ELISA were compared in measuring BRs and they determined that ELISA can facilitate the determination of brassinosteroid phytohormones in plant (Pradko et al., 2014). Besides, based on the simple, rapid, fidelity and cost-effective ELISA method, many endogenous hormones in higher plant were detected, such as IAA, gibberellin (GA), and methyl jasmonate (MeJA) (Chen et al., 2009; Wang et al., 2015, 2016; Wu et al., 2016).

According to previous studies, BR plays important role in leaf growth and development. The well-known example is that exogenous BR can affect the bending of the lamina joint of rice (Mori et al., 2002). In rice, *Dwarf1* gene was found to encode GTP-binding protein. The phenotype of its recessive mutant is dwarf (Li and Chory, 1997; Ross et al., 1997; Azpiroz et al., 1998). According to previous reports, the dwarf phenotype of *dwarf* mutants was associated with brassinosteroids and gibberellins (Hong et al., 2002). BR-deficient *dwarf 1*(*brd1*) mutant is a BR-related dwarf plant and the elongation of stem and leaves of which were reported to be repressed in rice (Hong et al., 2002). By contrast, exogenous BL stimulated the unrolling of leaf blades in wheat (Wada et al., 2014). In *Arabidopsis*, BR-deficient mutants appeared to have stunted shoot and small leaves (Nakaya et al., 2002). Here, the expression levels of genes involved in BR biosynthesis in leaf blades were higher than that in the root at the last three stages. The catabolism gene *DcCYP734A1* has the same expression trend in leaf blades of carrot plants. In addition, the fresh weight of aerial part of treated carrot plants (0.5 mg/L) was significantly higher than that of control. After treating by 24-EBL, genes *DcDWF4, DcCPD*, and *DcCYP85A2* in BRs biosynthesis pathway were down-regulated and catabolism gene *DcCYP734A1* was up-regulated at the concentration of 0.5 mg/L in the leaf blades. These results suggested that BRs may play important roles in carrot leaf blade growth.

BRs regulate plant growth and development through promoting cell elongation and division (Fridman and Savaldi-Goldstein, 2013). In *Arabidopsis*, the size and intercellular spaces of matured leaves of deetiolated2 (*det2)* and dwarf1 (*dwf1)* mutants were smaller than those of wild plants. These phenotypes of *det2* and *dwf1* mutants were demonstrated to be the result of inhibited elongation and division of cells in *Arabidopsis* (Nakaya et al., 2002). Similar results were also observed in rice (Hong et al., 2002). Plant height is an important index for plant growth. BR was reported to be a crucial factor for plant height (Hong et al., 2002). In soybean, *GmBRI1b* (*Glyma04g39160*) was a putative BR receptor and was found high similar to those of *AtBRI1* and pea *PsBRI1*. The petioles of *GmBRI1b* over-expression lines were longer than that of wild plants (Peng et al., 2016). Many BR-deficient mutants, such as *dwarf4* (*dwf4*), and *dwf7-1* in *Arabidopsis, dwarf1-1* in sorghum, exhibited dwarf phenotypes due to deficiency in BR biosynthesis (Choe and Feldmann, 1998; Cheon et al., 2010; Fridman and Savaldi-Goldstein, 2013; Petti et al., 2015). Here, the biosynthesis genes and catabolism genes showed higher expression levels at the first two stages which suggested that BRs may be involved in actively growing of petioles. In petioles of carrot plants, after being treated by exogenous BL, expression levels of most biosynthesis genes were inhibited. However, the catabolism gene *DcCYP734A1* was significantly up-regulated treated by exogenous BL at the concentration of 0.5 mg/L 24-EBL. Most signal transduction genes were also up-regulated when treated by 0.5 mg/L 24-EBL. Carrot plants treated by 24-EBL had increased height and aerial part weight. Meanwhile, the number of petioles in carrot plants increased after application of exogenous 24-EBL. All the above results suggested that application of exogenous 24-EBL promoted the growth of carrot petioles.

Carrot is a kind of root vegetable crop. The root of carrot is a good source of phytochemicals, such as carotenoids, anthocyanins, and other phenolic compounds. Till now, the brassinosteroid’s roles involved in carrot root growth and development are not clear. In *Arabidopsis*, application of exogenous brassinolide was reported to promote root elongation and lateral root germination (Gonzálezgarcía et al., 2011; Singh and Savaldigoldstein, 2015). However, BRs plays different roles in regulating nodule formation in different plant species. BRs can actively regulate the nodule numbers in pea, but function as an inhibitor in soybean nodule formation (Ferguson et al., 2005; Terakado et al., 2005). During the development of carrot, all biosynthesis and catabolism genes showed higher expression levels at the first two stages in root which suggested that BRs may be necessary for the early stage of carrot root growth and development. However, the expression levels of signal transduction genes do not show similar trend. After treatment, all the biosynthesis, catabolism and signal transduction genes were up-regulated. However, the root length and diameter of carrot plants treated with exogenous 24-EBL did not show significant changes in our study. Symons and his colleagues demonstrated that the long-distance transport of BRs cannot be detected in pea (Symons and Reid, 2004). In tomato, the long-distance transport of BRs is also lacking (Montoya et al., 2005). Moreover, BRs were thought to regulate root meristem size maintenance depending on their action site (Wei and Li, 2016). In *Arabidopsis*, the meristem size of root was reported to be controlled by BR signaling in the root epidermis (Gonzálezgarcía et al., 2011). In high plant, the extensive interactions were existed among different hormonal signaling pathways. Many kinds of hormones were involved in the regulation on plant growth and development (Nemhauser et al., 2006). In rice, the signaling pathways of auxin, ABA and GA3 controlling cell elongation are affected by other pathway involving brassinosteroids (Ephritikhine et al., 1999). In *Arabidopsis*, the growth of hypocotyls was reported to be controlled by auxin, ethylene and brassinosteroids (De et al., 2005). Ethylene is an important signal for the development of roots and was reported to enhance its inhibition of *Arabidopsis* roots by up-regulating auxin biosynthesis (Swarup et al., 2007). Moreover, the ethylene level in the primary roots of maize was also increased after application of exogenous brassinolide (Sun et al., 2002). In carrot plant, the growth and development of roots maybe controlled by BRs, and other plant hormones, auxin, ABA, GA3, and ethylene, and so on.

Phytohormone is important for plant, but the concentration of treatment should be appropriate. In *Arabidopsis*, low concentration of BL can promote the growth of root, but high concentrations will decrease it (Gonzálezgarcía et al., 2011). In present study, 24-EBL at 0.5 mg/L was proved to have a positive impact on carrot petiole growth. In conclusion, this study supports the idea that BRs play an important role in regulating plant height. Carrot plants exposed to exogenous 24-EBL were relatively higher than those treated with water alone. Exogenous 24-EBL can speed up the growth of petioles in carrots.

## Author Contributions

Conceived and designed the experiments: A-SX, FQ. Performed the experiments: FQ, G-LW, FW, Z-SX. Analyzed the data: FQ, G-LW. Contributed reagents/materials/analysis tools: A-SX. Wrote the paper: FQ. Revised the paper: A-SX, G-LW. All authors read and approved the final manuscript.

## Conflict of Interest Statement

The authors declare that the research was conducted in the absence of any commercial or financial relationships that could be construed as a potential conflict of interest.

## Footnotes

1 http://apiaceae.njau.edu.cn/carrotdb/index.php

## Abbreviations

24-EBL: 24-epibrassinolide
*ACTIN*: Actin 1 gene
BAK1: BRI1-associated receptor kinase1
BES1: BRI1-EMS-suppressor1
BIN2: bridging integrator 2
BRI1: Brassinosteroid insensitive 1
BRs: brassinosteroids
BSK1: BR-signaling kinase1
BSU1: BRI1 suppressor 1
BZR1: brassinazole resistant1
*CPD*: *CONSTITUTIVE PHOTOMORPHOGENESIS AND DWARFISM*
DAS: days after sowing
*DET2*: DE ETIOLATED2
*DWF1*: *DWARF1*
DWF4: *DWARF4*
*DWF7*: *DWARF7*
*TUB*: Tubulin beta-7 gene
qRT-PCR: quantitative reverse transcription PCR
